# Bone Ring Grafts Versus Non-Resorbable Membrane GBR for Vertical Ridge Augmentation: A Prospective Cohort Study

**DOI:** 10.3290/j.ohpd.c_2748

**Published:** 2026-06-30

**Authors:** Ammar Almarghlani, Hanin Alqurashy, Mohanad Alnahdi, Dalia Meisha, Ehab Azab, Hassan Abed, Rayan Sharka, Ali Alghamdi

**Affiliations:** a Ammar Almarghlani Associate Professor, Department of Periodontics, Faculty of Dentistry, King Abdul-Aziz University, Jeddah, Saudi Arabia. Conceptualization, methodology, validation, resources, supervision, project administration, read and agreed to the published version of the manuscript.; b Hanin Alqurashy General Dentist, Department of Periodontics, Faculty of Dentistry, King Abdul-Aziz University, Jeddah, Saudi Arabia. Conceptualization, methodology, data analysis, investigation, wrote original draft, read and agreed to the published version of the manuscript.; c Mohanad Alnahdi General Dentist, Department of Periodontics, Faculty of Dentistry, King Abdul-Aziz University, Jeddah, Saudi Arabia. Conceptualization, methodology, data analysis, investigation, wrote original draft, read and agreed to the published version of the manuscript.; d Dalia Meisha Associate Professor, Department of Dental Public Health, Faculty of Dentistry, King Abdul-Aziz University, Jeddah, Saudi Arabia. Software, validation, data analysis, wrote original draft, read and agreed to the published version of the manuscript.; e Ehab Azab Associate Professor, Department of Basic and Clinical Oral Science, Faculty of Dental Medicine, Umm Al-Qura University, Makkah, Saudi Arabia. Methodology, validation, resources, reviewed and edited the manuscript, read and agreed to the published version of the manuscript.; f Hassan Abed Associate Professor, Department of Basic and Clinical Oral Science, Faculty of Dental Medicine, Umm Al-Qura University, Makkah, Saudi Arabia. Data analysis, investigation, data curation, reviewed and edited the manuscript, read and agreed to the published version of the manuscript.; g Rayan Sharka Assistant Professor, Department of Oral and Maxillofacial Surgery, Faculty of Dental Medicine, Umm Al-Qura University, Makkah, Saudi Arabia. Data analysis, resources, data curation, wrote original draft, visualization, read and agreed to the published version of the manuscript.; h Ali Alghamdi Professor, Department of Periodontics, Faculty of Dentistry, King Abdul-Aziz University, Jeddah, Saudi Arabia. Conceptualization, validation, data curation, supervision, read and agreed to the published version of the manuscript. All authors have read and agreed to the published version of the manuscript.

**Keywords:** alveolar ridge reconstruction, bone-ring graft, guided bone regeneration, non-resorbable membrane, vertical bone augmentation.

## Abstract

**Purpose:**

Vertical alveolar bone defects often require advanced augmentation techniques to enable successful implant placement. The aim of this study was to compare short-term vertical bone gain following two approaches: the bone-ring graft technique and guided bone regeneration (GBR) using a non-resorbable membrane in patients with vertical alveolar deficiencies.

**Materials and Methods:**

A prospective cohort study was conducted involving 30 systemically healthy adult patients with posterior mandibular vertical bone defects measuring ≥4 mm. Participants were assigned to one of two treatment groups: Group A received the bone ring in conjunction with titanium implants and a resorbable collagen membrane; Group B was treated with allogeneic bone grafts and titanium-reinforced dense polytetrafluoroethylene (d-PTFE) membranes. Radiographic evaluations were performed at baseline, 1 week, 1 month, and 3 months postoperatively by cone-beam computed tomography (CBCT) and periapical radiographs. The primary outcome was vertical bone height gain. The data were analyzed using IBM SPSS Statistics Version 28.0.

**Results:**

Group A demonstrated statistically significantly greater vertical bone gain at all postoperative intervals: 9.1 mm at 1 week, 10.6 mm at 1 month, and 9.7 mm at 3 months, compared to 7.8 mm, 7.0 mm, and 6.9 mm, respectively, in Group B (p < 0.0001). Plaque index scores were comparable between groups, with no statistically significant differences observed in postoperative swelling or infection rates. However, membrane exposure was statistically significantly more frequent in Group B at all follow-up visits (p ≤ 0.02).

**Conclusion:**

The bone-ring graft technique was associated with greater vertical bone gain and a lower incidence of membrane exposure compared to GBR using a non-resorbable membrane. Further studies with longer follow-up are required to confirm these findings.

Vertical alveolar bone deficiencies are challenging in dental implantology, frequently compromising ideal implant positioning and long-term treatment outcomes.^19^ These defects are often attributable to chronic periodontitis, which induces sustained inflammation and progressive osseous degradation.^23^ Furthermore, postponement of grafting procedures following tooth extraction may exacerbate vertical bone loss, particularly when the alveolar ridge remains unrehabilitated for prolonged durations.^3,14^ Systemic conditions such as osteoporosis may further impair bone regeneration by altering bone metabolism and accelerating resorptive processes.^11^

In response to these challenges, vertical bone augmentation has become a central focus in implant dentistry.^25^ Guided bone regeneration (GBR) using non-resorbable membranes such as expanded polytetrafluoroethylene (e-PTFE) has gained widespread use for its ability to exclude soft tissue and support bone growth.^9^ The biological principle of GBR relies on the use of barrier membranes to exclude epithelial and connective tissue cells from the defect site, thereby allowing undisturbed bone regeneration.^24^ A key advantage of non-resorbable membranes is their mechanical durability; unlike resorbable alternatives, they retain structural integrity throughout the healing period, offering sustained support for osteogenesis.^16^ Nonetheless, their clinical success remains subject to debate, primarily due to complications such as membrane exposure and the associated risk of infection.^22^ Long-term evidence indicates that GBR can achieve stable horizontal bone augmentation, with reported gains of approximately 3–5 mm maintained over follow-up periods exceeding 10 years, provided that key biological and surgical principles such as appropriate case selection, space maintenance, and primary wound closure are respected.^4,7^

The clinical success of GBR is strongly influenced by the choice of barrier membrane and graft material.^4,26^ Titanium-reinforced nonresorbable membranes are frequently preferred for vertical ridge augmentation due to their superior mechanical stability and ability to maintain space against soft tissue pressure. However, these membranes are also associated with a higher risk of postoperative complications, particularly membrane exposure, which may compromise bone regeneration.^4,26^

In addition to membrane properties, soft tissue management and flap vascularity play a critical role in GBR outcomes. Fazekas et al^6^ demonstrated that GBR is associated with a temporary reduction in flap blood flow during early healing, particularly on the buccal aspect, although no consistent correlation was found between microcirculatory changes and hard tissue alterations. These findings emphasize the biological vulnerability of augmented sites and underscore the importance of atraumatic flap design and careful soft tissue handling to minimize ischemia and reduce the risk of postoperative complications, such as wound dehiscence and membrane exposure.^6^

Beyond soft tissue considerations, the biological behavior and resorption characteristics of bone graft materials are also decisive factors in augmentation outcomes. Emerging evidence suggests that grafts with lower resorption rates may provide improved volumetric stability in vertical defects, although they may prolong remodeling phases.^26^ These limitations have prompted interest in alternative augmentation strategies, such as the bone-ring graft technique, which provides intrinsic structural stability and allows simultaneous implant placement, potentially reducing treatment duration and complication risk in vertically deficient sites.^26^

The bone-ring graft technique has emerged as a promising alternative for managing vertical alveolar bone deficiencies.^20^ This novel technique allows simultaneous vertical ridge augmentation and implantation, leading to reduced treatment duration and enhanced patient outcomes.^1^ The procedure involves transferring a bone ring from a donor to augment diminutive alveolar ridges, facilitating the placement of dental implants in bone locations previously deemed inadequate.^1,13^ This technique entails excising an allograft bone ring that corresponds to the defect’s dimensions, and is advised for patients with vertical bone shortages who want to minimize conventional dental implants, as well as treatment duration and surgical interventions.

Despite the growing body of evidence supporting various techniques for vertical ridge augmentation, there are no reports of direct comparisons between bone-ring grafts and non-resorbable membranes in the literature. The bone-ring technique has demonstrated promising outcomes, particularly in enabling simultaneous implant placement and achieving substantial vertical bone gain.^20^ Similarly, GBR using non-resorbable membranes is well-established for its mechanical stability and predictable regenerative potential.^9^ However, the current body of evidence is largely limited to reviews and case reports, with few empirical clinical studies directly comparing their efficacy, complication rates, or long-term outcomes.^11,17,18,23^ This notable gap in current research underscores the need for studies that systematically evaluate the advantages and limitations of each technique in achieving predictable vertical bone regeneration. Therefore, this study was designed to compare the clinical effectiveness of the bone-ring graft technique and guided bone regeneration using a non-resorbable membrane as the primary vertical bone augmentation approach.

The secondary objective was to compare plaque index scores and complication rates between the two groups during the follow-up period. The research question was: Does the bone-ring graft technique result in superior vertical bone augmentation of alveolar defects compared to non-resorbable membranes? The study hypothesis was that the bone-ring graft technique would result in greater vertical bone augmentation compared to guided bone regeneration using a non-resorbable membrane. Accordingly, the null hypothesis stated that no statistically significant difference would be observed between the two techniques with respect to vertical bone gain.

## MATERIALS AND METHODS

### Study Setting and Design

This study was conducted at the Faculty of Dentistry of King Abdulaziz University, Jeddah, Saudi Arabia and was approved by the institutional review board (Approval No. 157-11-24).

A prospective cohort design was employed to evaluate the clinical effectiveness of the bone-ring graft method and guided bone regeneration using non-resorbable membranes. This study did not involve randomization. Participants were assigned to treatment groups based on their informed choice following a detailed clinical consultation to reflect routine clinical practice and to accommodate individual patient preferences, which may positively influence treatment adherence and satisfaction. All patients provided written informed consent before undergoing treatment. Data collection was standardized across groups to ensure consistency and enable a robust comparative analysis of clinical outcomes.

### Study Subjects

Healthy, adult patients aged 25 to 45 years were consecutively enrolled from those seeking implant therapy between January 1, 2025, and April 1, 2025, and presenting with a vertical peri-implant bone defect of at least 4 mm in the alveolar ridge in the mandibular posterior region. Exclusion criteria included the presence of systemic conditions such as uncontrolled diabetes or autoimmune disorders, bone diseases affecting healing, active smoking, prior bone grafting in the target area, a history of implant failure, use of medications known to interfere with bone regeneration, pregnancy, poor oral hygiene, known allergies to grafting materials, and inability to adhere to follow-up protocols.

The primary outcome of this study was vertical bone gain following augmentation. Sample size calculation was performed using G*Power statistical software based on the reported means and standard deviations of vertical bone gain from a previous study. Assuming a significance level (α) of 0.05 and a statistical power of 80%, the minimum required sample size was calculated as 13 participants per group, resulting in a total sample size of 26 subjects.

### Clinical Procedures

Preoperative CBCT scans were performed for digital planning and three-dimensional evaluation of alveolar bone volume and crestal height. All patients underwent standardized surgical protocols under local anesthesia.

**Fig 1:**
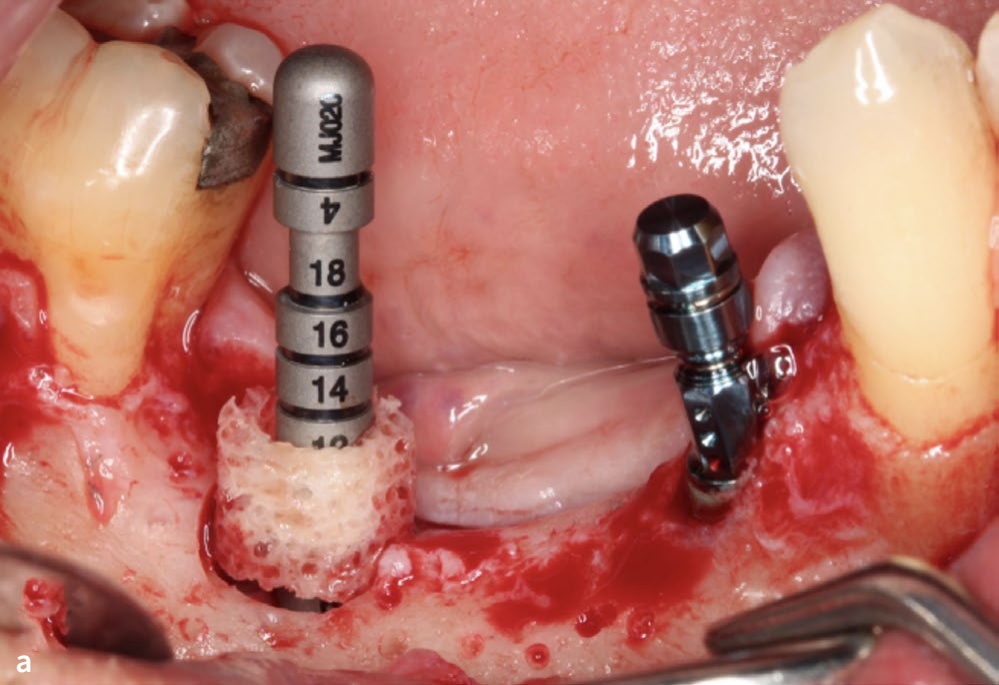

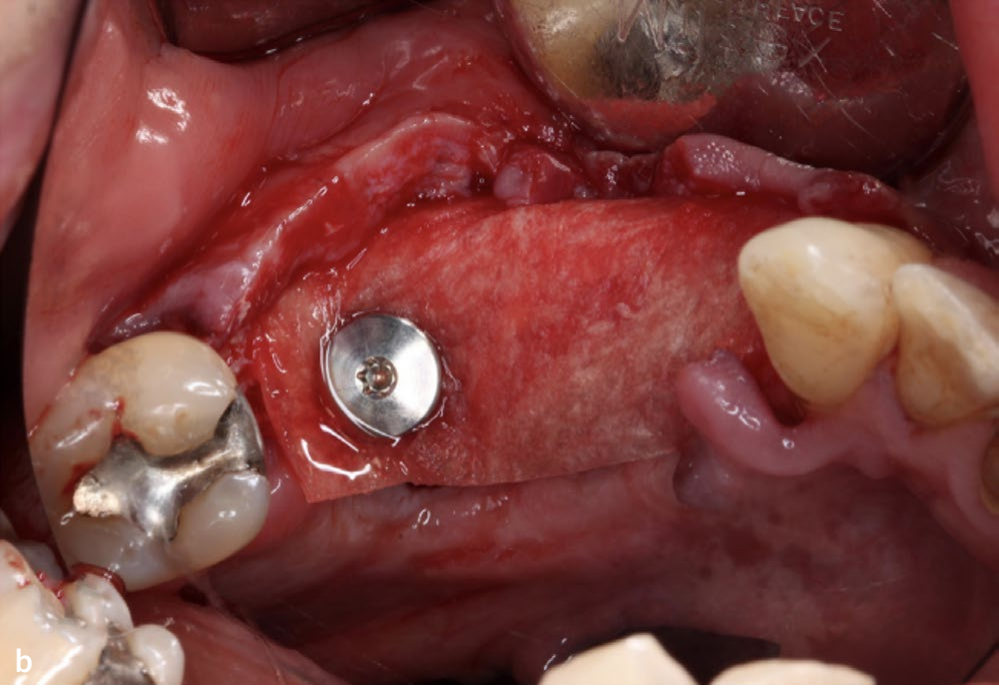
Bone ring grafting technique. a) Straumann maxgraft bone ring and Straumann titanium implants. b) A resorbable collagen membrane (ACE Resorb). In group B, guided bone regeneration was performed using titanium-reinforced nonresorbable PTFE membranes (Cytoplast Ti150) filled with hydrated allograft granules (see Fig 2a). The membrane was stabilized with osteosynthesis screws to ensure space maintenance. A resorbable collagen membrane was then placed over the PTFE membrane to support soft tissue healing (see Fig 2b). Flap elevation, mobilization, and wound closure were performed using the same protocol applied in group A.

In Group A, vertical bone augmentation was carried out using the bone-ring graft technique with allogeneic Straumann maxgraft bone rings (Straumann; Basel, Switzerland) with an outer diameter 6–7 mm), combined with Straumann titanium implants (Fig 1a). The height of the bone ring was adjusted intraoperatively according to the defect size. A mid-crestal incision with vertical releases was performed to elevate the flap and expose the alveolar ridge. Osteotomy preparation was guided by a surgical template and a trephine drill corresponding to the selected bone-ring size. The bone ring was positioned and stabilized, followed by implant placement to achieve primary stability. A resorbable collagen membrane (ACE Resorb, ACE Surgical Supply; Brockton, MA, USA) was placed over the graft and fixed with titanium pins to prevent soft tissue invasion (Fig 1b). Primary wound closure was achieved using periosteal releasing incisions.

**Fig 2:**
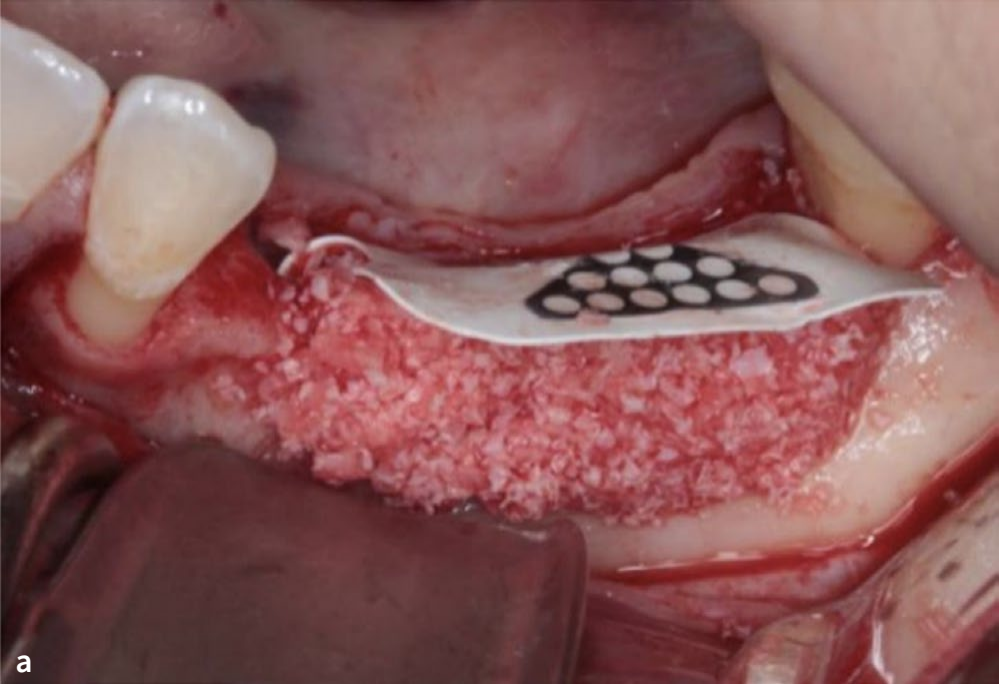

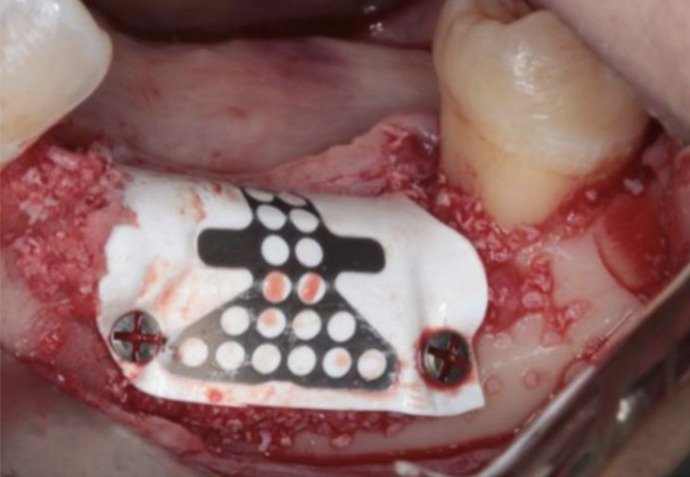
Guided bone regeneration (GBR) technique. a) GBR using titanium-reinforced non-resorbable PTFE membranes (Cytoplast Ti-150) filled with hydrated allograft granules. b) The membrane secured with osteosynthesis screws.

In group B, guided bone regeneration was performed using titanium‑reinforced non‑resorbable PTFE membranes (Cytoplast Ti‑150) filled with hydrated allograft granules (Fig 2a). The membrane was stabilized with osteosynthesis screws to ensure space maintenance. A resorbable collagen membrane was then placed over the PTFE membrane to support soft tissue healing (Fig 2b). Flap elevation, mobilization, and wound closure were performed using the same protocol applied in group A.

All patients received postoperative medication consisting of Augmentin (1 g) and Ibuprofen (600 mg) for infection control and pain management. Standardized periapical radiographs were obtained immediately after implant placement and at follow-up intervals of 1 week, 1 month, and 3 months, to monitor vertical bone changes. The second surgical procedure was planned for 4-6 weeks later, during which both the non-resorbable membranes and fixation caps were removed using a screwdriver, followed by suturing to complete the healing process. Plaque index was assessed at the implant site and on the buccal and lingual surfaces of the adjacent teeth using disclosing tablets at baseline and during followup visits. Vertical bone height was measured at the implant site as the distance from the implant shoulder to the most coronal point of the alveolar bone crest on the mesial and distal aspects, using standardized periapical radiographs.

**Fig 3:**
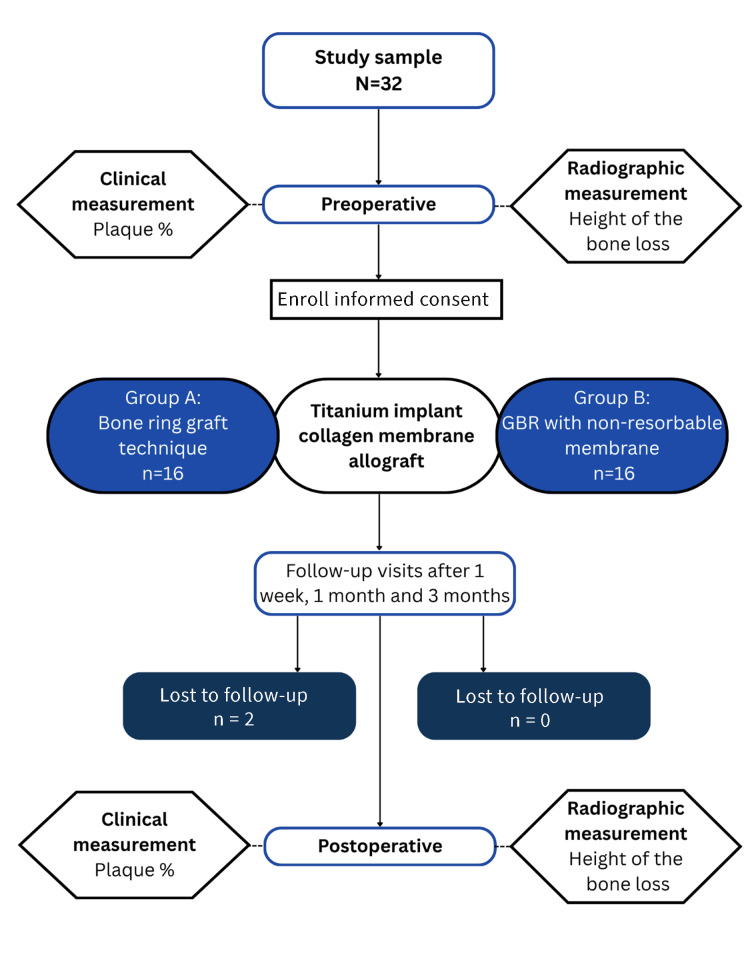
Study flow chart.

Figure 3 illustrates the study flowchart and the corresponding surgical protocols.

### Statistical Analysis

Statistical analyses were performed using IBM SPSS Statistics (Version 23, IBM; Armonk, NY, USA). Descriptive statistics were calculated, including means and standard deviations for continuous variables, and frequencies and percentages for categorical variables. The Shapiro-Wilk test was used to assess normality, and statistical significance was set at p > 0.05. Additionally, independent t-tests were used to compare quantitative variables such as plaque index and the height of the bone crest between the two treatment groups. Chi-squared tests were used to analyze categorical variables such as gender, age, site, tooth, gingival health, and complications, with significance set at p < 0.05.

## RESULTS 

A total of 32 patients were initially screened, with sixteen patients allocated to each group. Two patients from Group A discontinued follow-up after implant loading and were excluded from the final analysis.

### Patients’ Demographics

There were no statistically significant differences between groups at baseline in terms of age (33.1 ± 7.4 vs. 36.8 ± 8.9; p = 0.2), gender distribution (p = 0.2), implant site (p = 0.5), or tooth location (p = 0.9), confirming demographic comparability (Table 1).

**Table 1:** Demographic characteristics of the sample

	Group A (n = 14)	Group B (n = 16)	p-value
Age, mean ± SD	33.1 ± 7.4	36.8 ± 8.9	0.2
**Gender, n (%)** Male Female	8 (57.1%) 6 (42.9%)	5 (31.3%) 11 (68.8%)	0.2
**Site, n (%)** Lower left Lower right	6 (42.9%) 8 (57.1%)	9 (56.3%) 7 (43.8%)	0.5
**Tooth number** 35 36 45 46 47	**n (%)** 1 (7.1%) 5 (35.7%) 3 (21.4%) 4 (28.6%) 1 (7.1%)	**n (%)** 3 (18.8%) 6 (37.5%) 2 (12.5%) 4 (25.0%) 1 (6.3%)	0.9


### Pre-operative Clinical and Radiographic Measurements

There were no statistically significant differences in pre-operative clinical parameters (Table 2), with both groups classified as healthy (healthy vs gingivitis; p = 0.8) and having a similar plaque index (20.3% ± 7.1 vs. 20.6% ± 5.7; p = 0.9) and bone defect size (4.2 ± 0.9 mm vs 3.9 ± 1.0 mm; p = 0.4), confirming pre-operative clinical equivalence.

**Table 2:** Pre-operative clinical and radiographic measurements

	Group A (N = 14)	Group B (N = 16)	p-value
**Status** Healthy gingiva Gingivitis	5 (50.0%) 9 (45.0%)	5 (50.0%) 11 (55.0%)	0.8
Pre-operative plaque index (%), mean ± SD	20.3% ± 7.1	20.6% ± 5.7	0.9
Preoperative bone defect, mean ± SD	4.2 ± 0.9	3.9 ± 1.0	0.4


### Vertical Bone Height

Post-operative analysis demonstrated a statistically significant vertical bone height advantage for Group A (Table 3), with patients in this group demonstrating a greater bone height at one week (p = 0.04), one month (p < 0.0001), and three months (p < 0.0001) compared to Group B.

**Table 3:** Bone crest height as measured via CT

Follow-up period	Bone crest height (mm)		p-value
	Group A	Group B	
1 week	9.1 ±1.7	7.8 ± 1.6	0.04*
1 month	10.6 ± 1.6	7.0 ± 1.2	<0.0001*
3 months	9.7 ± 0.9	6.9 ± 1.0	<0.0001*
*Statistically significant at p < 0.05.			

### Plaque Index Outcomes and Clinical Complications

Plaque index outcomes were not statistically different between groups (Table 4).

**Table 4:** Post-operative plaque index

Follow-up period	Post-operative plaque index (%)		p-value
	Group A	Group B	
1 month	18.0%± 6.1	20.6% ± 7.1	0.3
3 months	21.7% ± 9.4	25.3% ± 11.9	0.4


Membrane exposure was statistically significantly more frequent in Group B at one week (p = 0.02), one month (p = 0.01), and three months (p = 0.01). No statistically significant differences were observed between groups in terms of swelling, infection, or failure rates at any time point (p > 0.05) (Table 5).

**Table 5:** Clinical complications

	Group A	Group B	p-value
**After 1 week**			
Swelling	5 (33.3%)	10 (66.7%)	0.1
Membrane exposure	3 (23.1%)	10 (76.9%)	0.02*
Infection	4 (44.4%)	5 (55.6%)	0.9
**After 1 month**			
Swelling	–	–	–
Membrane exposure	1 (11.1%)	8 (88.9%)	0.01*
Infection	1 (20.0%)	4 (80.0%)	0.2
**After 3 months**			
Swelling	–	–	–
Membrane exposure	1 (11.2%)	8 (88.9%)	0.01*
Infection	0	3 (100.0%)	0.09
Failure	0	3 (100.0%)	0.09


## DISCUSSION

This study aimed to evaluate whether the bone-ring graft technique offers superior vertical bone augmentation alveolar bone defects. Radiographic and clinical assessments indicated that the bone-ring graft group consistently demonstrated greater vertical bone gain across all follow-up periods compared to the group treated with non-resorbable membranes. These outcomes directly address the central research question and substantiate the hypothesis that immediate implant integration combined with enhanced structural stability statistically significantly improves vertical bone augmentation.

The results align with established theoretical explanations in bone regeneration, with the superior performance of the bone-ring graft technique attributed to its capacity to provide immediate mechanical support and volumetric stability.^1,20^ The observed differences between the two groups may also be influenced by the inherent properties of the graft materials used. The bone-ring graft represents a solid, three-dimensional structure that provides immediate mechanical stability and maintains the augmented space throughout healing. In contrast, hydrated allograft granules rely primarily on membrane support for space maintenance and are more susceptible to micromovement and volume reduction, particularly in vertical defects. The rigid configuration of the bone ring allows simultaneous implant placement and facilitates load transfer to the native bone, which may promote more predictable bone regeneration. These material-related differences likely contributed to the greater vertical bone gain observed in the bone ring group. This structural advantage facilitates more effective bone healing compared to traditional non-resorbable membrane approaches.^1,20^ The findings of the present study are consistent with those reported in a randomized controlled trial comparing GBR using non-resorbable d-PTFE titanium-reinforced membranes and titanium meshes covered with cross-linked collagen membranes in the posterior mandible.^5^ This study demonstrated comparable outcomes between the two approaches in terms of complication rates, vertical bone gain, and implant stability.^5^ These parallels reinforce the clinical viability of both techniques for vertical ridge augmentation in challenging posterior mandibular sites.

Moreover, the outcomes of the present study align with those reported in a clinical investigation evaluating vertical ridge augmentation and simultaneous implant placement using the allograft bone ring technique.^15^ The authors reported a success rate of 97.5% over a 12-month follow-up period and an average vertical graft shrinkage of 8.6% for 81 implants placed in 51 patients.^15^ These comparable findings collectively demonstrate favorable implant stability and vertical bone gain using the bone-ring graft approach. The consistency between studies reinforces the reliability of this technique in managing large vertical defects while minimizing treatment time and donor site morbidity.

When compared with existing literature, the findings of the present study are largely consistent with previously reported outcomes for both GBR and bone-ring–based augmentation techniques. Previous studies evaluating GBR with nonresorbable membranes have reported predictable bone gain but also highlighted membrane exposure as a frequent complication, particularly in vertical defects.^4,8^ Similarly, clinical reports on the bone-ring graft technique have demonstrated favorable early bone gain and implant stability when simultaneous implantation is performed.^12,20^ The greater vertical bone gain observed in the bone-ring group in the present study is in agreement with these reports and may be explained by the inherent structural stability of the ring graft compared with particulate grafts supported by membranes. Differences in complication profiles between studies may be related to variations in defect morphology, flap design, and followup duration.^4,8^

The absence of statistically significant differences in plaque index between the bone-ring and GBR (non-resorbable membrane) groups in our study aligns with findings from other implant-related research. For instance, Attia et al^2^ conducted a long-term evaluation of dental implants placed in augmented bone treated with platelet-rich plasma (PRP) following sinus lift surgery using autologous iliac crest bone grafts, reporting no statistically significant differences between the PRP and control groups across several clinical parameters, including plaque index and other periodontal parameters. Interestingly, while the PRP group showed inferior outcomes in 64% of the evaluated parameters, these differences did not reach statistical significance. Also, another retrospective study involving over 760 implants compared outcomes between implants placed with and without bone augmentation, including techniques such as GBR, ridge splitting, and onlay grafting.^21^ The researchers found that plaque index scores were statistically comparable across all groups, regardless of whether bone augmentation was performed.^21^ This suggests that the presence or absence of augmentation procedures does not statistically significantly influence plaque accumulation around implants, especially when patients follow standardized oral hygiene protocols. The similarity in plaque index outcomes across different augmentation methods indicates that plaque control is more dependent on patient behavior and maintenance than on the surgical technique or materials used. It also supports the clinical relevance of focusing on patient education and follow-up care to maintain peri-implant health, rather than expecting differences based solely on augmentation type.

In the present study, the statistically significantly higher incidence of membrane exposure in Group B raises important clinical concerns regarding the predictability and overall success of GBR procedures. This observation is supported by evidence from a systematic review and meta-analysis, which demonstrated that membrane exposure has a markedly negative impact on bone augmentation outcomes.^8^ Specifically, sites without exposure achieved substantially greater horizontal bone gain and more effective defect reduction at peri-implant locations. The analysis revealed that exposed membranes were associated with a 74% reduction in horizontal bone gain and a 27% decrease in dehiscence resolution compared to sites with intact membranes.^8^ Also, another systematic review found that healing complications, including membrane exposure, are associated with a notable reduction in vertical bone gain following GBR procedures.^22^ Specifically, sites with membrane exposure achieved approximately 35–38% less vertical bone regeneration compared to those with uneventful healing. While the overall incidence of such complications was relatively low, their occurrence statistically significantly compromised the effectiveness of the augmentation.^22^ However, these studies have also emphasized that vertical ridge augmentation remains more techniquesensitive and is associated with a higher risk of complications compared with horizontal procedures, particularly when rigid nonresorbable membranes are used.^4,26^ Membrane exposure has been consistently identified as a major limiting factor affecting the predictability of regenerated bone, which is consistent with the higher exposure rate observed in the GBR group in the present study.^26^ Furthermore, recent investigations into flap microcirculation have highlighted the biological vulnerability of augmented sites during the early healing phase, underscoring the importance of atraumatic flap management and tensionfree wound closure.^6^ Although direct correlations between flap blood flow and hard tissue outcomes remain inconclusive, impaired early softtissue healing may contribute to wound dehiscence and membrane exposure, potentially compromising regeneration. The reduced exposure rate observed in the bone-ring group may therefore be partly attributed to the intrinsic structural stability of the graft and the reduced reliance on rigid membranebased space maintenance.

Therefore, the high exposure rate in Group B may reflect procedural or anatomical challenges that warrant further investigation. Future studies should aim to identify predictive factors for membrane exposure and explore strategies such as improved flap design or alternative membrane materials that could enhance healing outcomes and maximize bone regeneration.

This study has several limitations that may influence the interpretation and generalizability of the results. The relatively small sample may have impacted statistical power. Additionally, the relatively short follow-up period restricts our ability to draw firm conclusions regarding the long-term stability of regenerated bone and the durability of clinical outcomes. The absence of histological analysis further limits insight into the biological mechanisms driving osteogenesis, leaving questions about the quality and maturation of the newly formed bone unanswered. Furthermore, standardized pre- and post-operative clinical and radiographic photographs were not available for all patients, which limited the ability to visually document surgical outcomes beyond quantitative radiographic measurements. Despite these limitations, the study provides clinically relevant comparative data on early healing outcomes in vertical bone augmentation.

## CONCLUSION

This study provides short-term clinical evidence suggesting that the bone-ring graft technique is associated with greater vertical bone gain and a lower incidence of membrane exposure compared to guided bone regeneration using a non-resorbable membrane. These findings reflect differences observed during the early healing phase and should be interpreted within the limitations of short follow-up period. While the results indicate potential advantages of the bone-ring graft approach in terms of early vertical augmentation and complication profile, longer-term studies are required to confirm the stability and clinical relevance of these outcomes over time.

## References

[ref1] Ali SS, Wankhede AN (2021). Prerequisite for successful implant placement with a comparative study on novel bone ring technique: a review. J Pharm Res Int.

[ref2] Attia S, Narberhaus C, Schaaf H, Streckbein P, Pons-Kühnemann J, Schmitt C (2020). Long-Term influence of platelet-rich plasma (prp) on dental implants after maxillary augmentation: retrospective clinical and radiological outcomes of a randomized controlled clinical trial. J Clin Med.

[ref3] Avila-Ortiz G, Elangovan S, Kramer KWO, Blanchette D, Dawson DV (2014). Effect of alveolar ridge preservation after tooth extraction: a systematic review and meta-analysis. J Dent Res.

[ref5] Cucchi A, Vignudelli E, Napolitano A, Marchetti C, Corinaldesi G (2017). Evaluation of complication rates and vertical bone gain after guided bone regeneration with non-resorbable membranes versus titanium meshes and resorbable membranes. A randomized clinical trial. Clin Implant Dent Relat Res.

[ref6] Fazekas R, Molnár B, Sólyom E, Somodi K, Palkovics D, Molnár E, Sculean A, Vág J (2024). Relationship between flap microcirculation and hard tissue changes following alveolar ridge augmentation: a prospective case series. Quintessence Int.

[ref7] Fu JH, Choo HJS, Ong DS, Kwek H (2026). Long-term stability of horizontal bone augmentation at implant sites. Periodontol 2000.

[ref8] Garcia J, Dodge A, Luepke P, Wang HL, Kapila Y, Lin GH (2018). Effect of membrane exposure on guided bone regeneration: A systematic review and meta-analysis. Clin Oral Implants Res.

[ref9] Guedes EVB, Figueira MMM, Figueira LCG, Jesus ORV de, Araújo ME de (2024). Guided Bone Regeneration: Descriptive and Retrospective Study Between PRF Membranes and PTFE-e. Braz J Implantol Health Sci.

[ref10] Li Y, Ling J, Jiang Q (2021). Inflammasomes in Alveolar Bone Loss. Front Immunol.

[ref11] Liu J, Kerns DG (2014). Mechanisms of guided bone regeneration: a review. Open Dent J.

[ref12] Markou N, Pepelassi E, Vavouraki H, Stamatakis HC, Nikolopoulos G, Vrotsos I (2009). Treatment of periodontal endosseous defects with platelet-rich plasma alone or in combination with demineralized freeze-dried bone allograft: a comparative clinical trial. J Periodontol.

[ref13] Miller RJ, Korn RJ, Miller RJ (2020). Indications for simultaneous implantation and bone augmentation using the allograft bone ring technique. int j periodontics restorative dent.

[ref14] Morjaria KR, Wilson R, Palmer RM (2014). Bone Healing after Tooth Extraction with or without an Intervention: A systematic review of randomized controlled trials. Clin Implant Dent Relat Res.

[ref15] Nord T, Yüksel O, Grimm WD, Giesenhagen B (2019). One-stage vertical ridge augmentation and dental implantation with allograft bonerings: results 1 year after surgery. J Oral Implantol.

[ref16] Patil S, Bhandi S, Bakri MMH, Albar DH, Alzahrani KJ, Al-Ghamdi MS (2023). Evaluation of efficacy of non-resorbable membranes compared to resorbable membranes in patients undergoing guided bone regeneration. Heliyon.

[ref17] Rakhmatia YD, Ayukawa Y, Furuhashi A, Koyano K (2013). Current barrier membranes: Titanium mesh and other membranes for guided bone regeneration in dental applications. J Prosthodont Res.

[ref18] Ren Y, Fan L, Alkildani S, Liu L, Emmert S, Najman S (2022). Barrier membranes for guided bone regeneration (GBR): a focus on recent advances in collagen membranes. Int J Mol Sci.

[ref20] Sáez-Alcaide LM, Brinkmann JCB, Sánchez-Labrador L, Pérez-González F, Molinero-Mourelle P, López-Quiles J (2020). Effectiveness of the bone ring technique and simultaneous implant placement for vertical ridge augmentation: a systematic review. Int J Implant Dent.

[ref21] Tatli U, Cavana A, Tukel HC, Benlidayi ME (2025). Effects of bone augmentation on implant success and survival: a retrospective analysis with 6-year mean follow-up. Clin Implant Dent Relat Res.

[ref22] Tay JRH, Ng E, Lu XJ, Lai WMC (2022). Healing complications and their detrimental effects on bone gain in vertical-guided bone regeneration: A systematic review and meta-analysis. Clin Implant Dent Relat Res.

[ref23] Tsuchida S, Nakayama T (2023). Recent clinical treatment and basic research on the alveolar bone. Biomedicines.

[ref24] Yang Z, Wu C, Shi H, Luo X, Sun H, Wang Q (2022). Advances in barrier membranes for guided bone regeneration techniques. Front Bioeng Biotechnol.

[ref25] Zhang M, Zhou Z, Yun J, Liu R, Li J, Chen Y (2022). Effect of different membranes on vertical bone regeneration: a systematic review and network meta-analysis. BioMed Res Int.

[ref26] Zhan H, Shi R, Ni H, Li H, Yuan C, Lin K, Sculean A, Miron RJ (2025). Functional requirements for guided bone regeneration/guided tissue regeneration membrane design: Progress and challenges. Periodontol 2000.

